# Chromatin-driven *de novo *discovery of DNA binding motifs in the human malaria parasite

**DOI:** 10.1186/1471-2164-12-601

**Published:** 2011-12-13

**Authors:** Elena Y Harris, Nadia Ponts, Karine G Le Roch, Stefano Lonardi

**Affiliations:** 1 Department of Cell Biology and Neuroscience, University of California, Riverside (CA) 92521 USA; 2 Department of Computer Science and Engineering, University of California, Riverside (CA) 92521 USA

## Abstract

**Background:**

Despite extensive efforts to discover transcription factors and their binding sites in the human malaria parasite *Plasmodium falciparum*, only a few transcription factor binding motifs have been experimentally validated to date. As a consequence, gene regulation in *P. falciparum *is still poorly understood. There is now evidence that the chromatin architecture plays an important role in transcriptional control in malaria.

**Results:**

We propose a methodology for discovering *cis*-regulatory elements that uses for the first time *exclusively *dynamic chromatin remodeling data. Our method employs nucleosome positioning data collected at seven time points during the erythrocytic cycle of *P. falciparum *to discover putative DNA binding motifs and their transcription factor binding sites along with their associated clusters of target genes. Our approach results in 129 putative binding motifs within the promoter region of known genes. About 75% of those are novel, the remaining being highly similar to experimentally validated binding motifs. About half of the binding motifs reported show statistically significant enrichment in functional gene sets and strong positional bias in the promoter region.

**Conclusion:**

Experimental results establish the principle that dynamic chromatin remodeling data can be used *in lieu *of gene expression data to discover binding motifs and their transcription factor binding sites. Our approach can be applied using only dynamic nucleosome positioning data, independent from any knowledge of gene function or expression.

## Background

One of the major challenges in molecular biology is to characterize the mechanisms governing the regulation of transcription. Mechanisms of regulation can be broadly classified in three classes: (1) interaction of a control factor with DNA, (2) interaction of a control factor with the transcriptional complex, and (3) epigenetic factors. In this paper we are interested in elucidating mechanisms that belong to the first class, in which *transcription factor *proteins modulate expression levels by binding to one or more specific sites in the promoter region of a gene. The problem of identifying *in silico *transcription factor binding sites (TFBS) has been studied intensively. As a result a variety of algorithms and tools have been developed (see [1] for a review). Typically, these methodologies involve obtaining a set of genes which are known to be co-expressed or functionally-related and searching for common (over-represented) short "motifs" in their promoter regions. The underlying hypothesis is that co-expressed or functionally-related genes are expected to share common TFBS.

Algorithms for motif discovery can be *enumerative *or *alignment-based*. Enumerative techniques involve the enumeration of all the possible motifs in the promoters, the assignment of an appropriate score based on probabilistic models, and a criteria to select the most statistically significant motifs. Alignment-based methods use probabilistic modeling and combinatorial optimization, *e.g.*, expectation maximization or Gibbs sampling, to identify sequence patterns that are over-represented in the context of the promoter regions. Commonly used tools for motif discovery include MEME [2], Weeder [3], Gibbs Motif Sampler [4] and AlignACE [5]. In general, the discovery of very short or highly degenerated motifs remains statistically challenging. Therefore, these tools have only a limited success when used alone, especially when the ~80% AT-rich genome of the human malaria parasite is considered. In response to an urgent need to understand how *P. falciparum *regulates its genes, several *ad-hoc *techniques to discover *cis*-regulatory elements have been developed specifically for the malaria parasite. Young *et al*.'s Gene Enrichment Motif Searching (GEMS) [6] uses a hypergeometric-based scoring scheme and a position-specific weight matrix optimization procedure to identify putative motifs. The input to GEMS are twenty-one clusters of functionally related or co-expressed genes in *P. falciparum*. The ouput is 34 putative TFBS in promoter sequences and 21 TFBS in introns-derived sequences. The method proposed by Wu *et al*. [7] compares evolutionarily related species of *Plasmodium *and uses orthologous sequences to identify conserved TFBS. The method identified 38 TFBS that partially overlap previously reported putative TFBS in *P. falciparum *[2,8]. Elemento *et al*. [9,10] propose an algorithm called Finding Informative Regulatory Elements (FIRE) that measures the mutual information between sequences and gene expression profiles, which is used to select the most statistically significant motifs. While most of the experiments are carried out in *Saccharomyces cerevisiae*, the authors report 21 putative TFBS in *P. falciparum*. Iengar and Joshi [11] combined the strenght of MEME [2], AlignACE [5] and Weeder [3], to identify putative TFBS in promoters of *P. falciparum *co-expressed genes. The authors used strict cut-offs and selected only motifs that were found by all three software tools. This study resulted in 27 sets of putative TFBS.

The success of computational TFBS discovery methods critically depends on the *a priori *knowledge about which genes are co-regulated or functionally-related and therefore more likely to share common TFBS. Carrying out this step can be challenging because (1) gene functional annotation are often incomplete or inaccurate, (2) gene expression profiles are often limited to a subset of the genes or specific events during the cell cycle and (3) genome-wide expression data can be incomplete. For instance, *P. falciparum *has 5418 protein-coding genes of which about 2500 have no known function and only ~3200 have stable and constitutive expression profile. Furthermore, gene clustering rely on the measurement of mRNA steady state levels that are not a direct measure of transcriptional activity but reflect both mechanisms of transcriptional and post-transcriptional regulation. As a consequence, the resulting clusters are likely to be incomplete or incorrect.

Recent alternative approaches that exploit the chromatin structure information to identify TFBS are based on the observation that active TFBS are usually nucleosome-depleted. Nucleosome occupancy data has already shown to improve the discovery of TFBS employed in a few model organisms (*e.g.*, [12-15]). Nucleosome information is typically used in combination with sequence sets generated by ChIP experiments and occasionally mRNA expression profiles rather than alone. Collecting all nucleosome-depleted regions of the genome would indeed generate a very high number of sequence sets without transcription-related specificity. To circumvent this problem, we propose to use *dynamic *changes of nucleosome occupancy (*e.g.*, across time, such as the duration of a cell cycle) to build sets of genes that are likely to be co-regulated. We demonstrate the power of our approach in the context of the search for potential TFBS in *P. falciparum*'s genome using nucleosome positioning data obtained at seven time points during the erythrocytic asexual cycle. This approach is general, and can be applied to any organism for which nucleosome occupancy data at various time points or under varying conditions are available. Here, we identify 129 potential DNA binding motifs in the human malaria parasite's genome, most of them being novel. These results represent a major resource for the human malaria parasite, with significant implications for future investigations.

## Results and Discussion

### Methodology outline

In a previous analysis of the chromatin architecture in *P. falciparum *[16], we used FAIRE (formaldehyde-assisted isolation of regulatory elements) coupled with next generation sequencing, or FAIRE-seq, to study the variations of nucleosome occupancy across its intra-erythrocytic cycle. Samples of *P. falciparum*-infected erythrocytes were collected with six-hours increment for 36 hours (seven time points), which is the duration of one cycle of growth, replication, and maturation into multiplied invasive parasites. The parasites' chromatin status at all seven time points was analyzed by FAIRE-seq. Briefly, the method involves the chemical cross-linking of the chromatin followed by shearing, protein-free DNA purification, and sequencing. FAIRE-seq therefore isolates and reveals nucleosome-free regions of the genome. In general, we found that nucleosomes are more abundant within malaria gene bodies whereas promoters are relatively nucleosome-depleted (*i.e.*, with high FAIRE-seq read coverage). In that context, we analyzed the variations of chromatin availability (*i.e.*, the variations of FAIRE-seq read coverage) at the *loci *of two validated TFBS that are specific to apicomplexan AP2-related transcription factors [17]. We observed that the chromatin availability of these TFBS varies significantly across time [16]; the presence/absence of nucleosomes masks/reveals the TFBS and thereby modulates the binding of the ApiAP2 transcription factors. These elements indicate that one should be looking for TFBS within the regions of the genome with variable FAIRE-seq coverage. Here, we analyze the regions of the *P. falciparum *genome covered by FAIRE-seq sequenced reads with the highest variance of FAIRE-seq coverage across the seven time points of the erythrocytic cycle - *i.e.*, nucleosome-depleted regions that are potentially accessible to transcription factors in a cycle-dependent manner - to discover novel putative TFBS.

**Figure 1:**
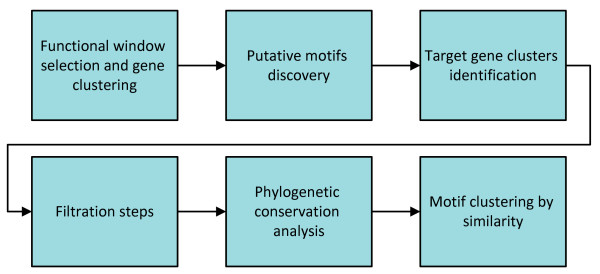
**Overview of the proposed methology to discover TFBS using FAIRE-seq read coverages**.

**Figure 2:**
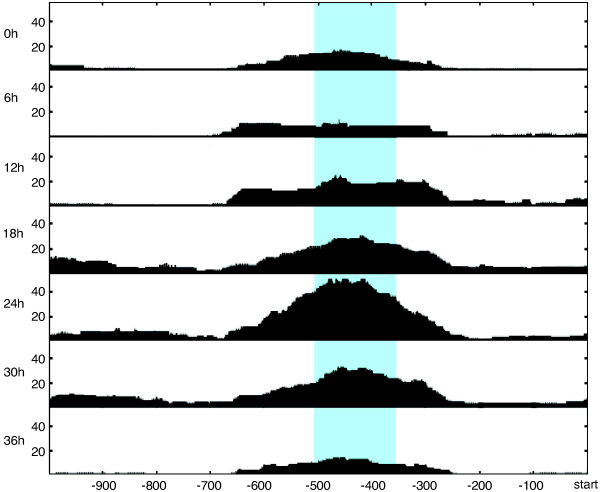
**FAIRE-seq coverage and functional window for a *P. falciparum *gene**. Each box shows FAIRE-seq coverage (black) of the region at a given time point (0 h to 36 h with six hours increments). The functional window with the highest variance of FAIRE-seq is shown in light blue.

Figure 1 illustrates an overview of our method (details can be found in the Methods section). First, we define the *functional window *of a gene as the nucleosome-sized region within 1000 bp upstream of the start codon (regions containing promoters) with the highest variance of FAIRE-seq coverage across the seven time points (see Figure 2 for an example). The choice of the functional window is completely independent from the sequence content. In fact, the nucleotide distribution inside the functional windows and in the rest of the promoters are highly similar (the proportion of As, Cs, Gs and Ts inside the functional windows compared to the rest of the promoters respectively are: 0.416 vs. 0.422; 0.064 vs. 0.068; 0.064 vs. 0.070; 0.456 vs. 0.440).

The FAIRE-seq coverage within the functional window is then averaged for each time point, creating a seven-point chromatin availability profile for the considered gene These profiles are used for *k*-means clustering of the genes. Using *k *= 15, we obtained clusters of 33 to 841 genes, for an average of 364 genes per cluster (data not shown). The sequence sets from the functional windows within the same cluster are used as input for the discovery of putative TFBS, hereafter called *motifs*. Since the only experimentally-validated motifs for *P. falciparum *are either six or eight bp-long, we restricted the present analysis to motifs of six, seven and eight nucleotides in length. Our method can however be used with motifs of any length.

For each cluster, all possible motifs of size six to eight nucleotides in length were searched within the functional windows of each gene. Frequencies of each motif were then modelled according to a hypergeometric probability distribution that measures the chances that the frequency of a given motif would be observed if the input sequences would have been selected randomly. The *hypergeometric enrichment score *or HES captures the statistical significance of the over-representation of a given motif in a given group (e.g., significance threshold set at HES = 2 ⇔ *p *= 0.01). Motifs and their variants were selected according to their HES values. This step selected 2727 6-mers, 8813 7-mers, and 19,435 8-mers in the *P. falciparum *genome.

**Figure 3:**
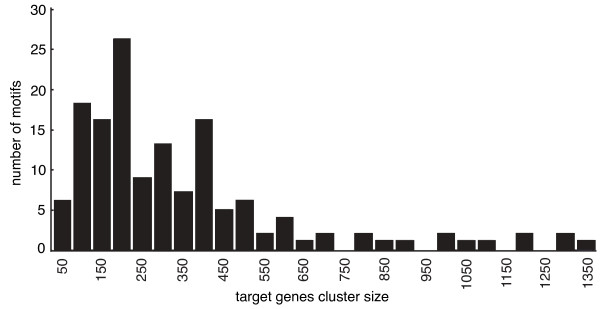
**Distribution of motifs frequencies according to target gene cluster sizes**. The average cluster size is 139 genes (standard deviation = 279; min = 41; max = 1333).

In the next refinement step, we examined the representation of each selected motif in the genome. For each motif, we selected all the genes with at least one occurrence of the motif within their functional windows. This set of genes is the *target gene cluster *of a given motif. Figure 3 shows the distribution of motifs representation according to the sizes of the target gene clusters. The majority of motifs are found within target gene clusters with sizes ranging from 100 to 400. The average cluster size is 139 genes with a standard deviation of 279. These observations are consistent with the fact that the distance between genes of larger clusters is higher than the one measured for clusters more restricted in size. Such big clusters are likely to contain a more diluted and noisy information and thus to contain a lesser amount of potential motifs. We proceeded to another round of motif identification using our HES-based method applied to the functional windows of genes within their target clusters. In order to remove spurious motifs three filtering steps are carried out. First, motifs with a HES lower than the average HES for all motifs of the same length were excluded. The average values of HES for 6-, 7- and 8-mers were 121.9, 88.19 and 73.16 respectively. For our data set, this requirement on HES is significantly more stringent than application of the Bonferroni correction coefficient for multiple testing. Then we used phylogenetic conservation information. Four other *Plasmodium *species closely related to *P. falciparum *were considered in our analysis: *Plasmodium vivax *(human host), *Plasmodium berghei*, *Plasmodium chabaudi*, and *Plasmodium yoelii *(rodent hosts). A motif was retained for downstream analysis when its HES in corresponding orthologous genes was at least two (*i.e.*, *p-value *≤ 0.01) in at least one of the four orthologous species. To ensure that high orthologous HES were not due to a random chance, we applied a randomized analysis of orthologous HES that further reduced the list of potential candidates for true motifs. After the filtration step, a total of 227 6-mers, 125 7-mers and 217 8-mers remained.

Finally, since motifs are usually degenerated to various extents, they were regrouped according to their similarities measured by the *Tanimoto *distance [3,6] and the Pearson coefficient of position weight matrix (PWM) [18]. Ultimately, the final list of degenerated motifs consisted of 129 putative sets, *i.e.*, 21 6-mers, 46 7-mers, and 62 8-mers (this lists of motifs can be found in Additional File 1). Our tool can provide different representations for a motif: as the list of mutants, as a position weight matrix (PWM), as a sequence logo or as a regular expression. This flexibility allows users to make an informed decision about which particular sequence of a motif to select in experimental validation.

We evaluated the performances of our method by comparison with previously published work. We analyzed the distribution of our motifs within the *P. falciparum *genome, looking for positional biases relative to transcription start sites and predicted promoters, and for enrichment in functional sets of genes built from gene ontology and gene expression profiles information.

### Comparison with previously proposed motifs

**Figure 4:**
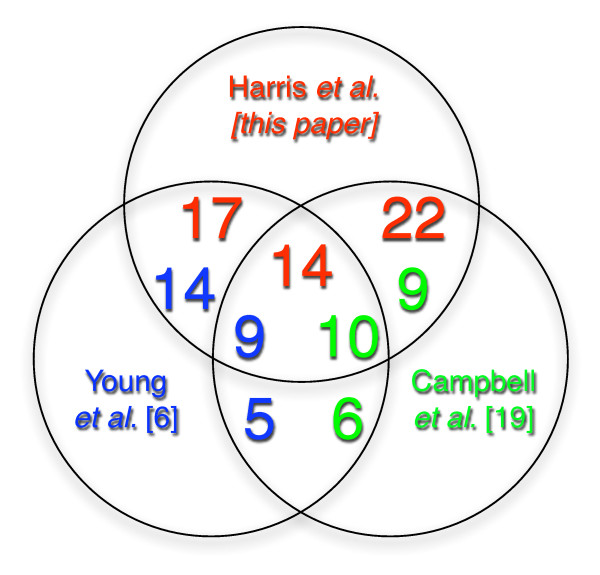
**Venn diagram of multi-method data comparison**. Overlap between motif discovery results obtained using the proposed method, Young *et al*. [6], and Campbell *et al*. [19].

We compared our 129 candidate motifs with previously validated motifs [6,17,19,20]. More specifically, we examined the ApiAP2 transcription factor binding motifs for PF14_0633 (TGCATGCA) and PFF0200c (GTGCAC) reported in [17], and the motif NGGTGCA associated with the gene invasion cluster [6,20]. A position weight matrix (PWM) was built for each of our motif sets and the various occurrences of the previously published motifs [17]. The Pearson coefficient of corresponding PWMs was used as similarity metric. Results are summarized in Table 1. The top-scoring motif in our list had a similarity score of 0.9969 with PFF0200c [17]. Another of the motifs in our list matched the motif NGGTGCA [6] with a similarity score of 0.833. The most similar motif to PF14_0633 in our list was TATGCAT with the similarity score of 0.704. We further compared our list of 129 motif sets with the full list of 50 candidate motifs reported in [6] and the 23 candidate motifs (positional matrices from [19]). The comparison was carried out by computing the Pearson coefficient between the corresponding PWM and reporting only pairs that exceeded 0.75 similarity. We found that 30 of our motif sets were highly similar to 23 of the motifs from [6], and 35 of our motifs were similar to 19 of the motifs from [19] (Figure 4). The fact that our list of motifs contains biologically validated motifs and that several motifs are shared with previous studies validates the exclusive use of chromatin structural change via FAIRE-seq for the discovery of TFBS in an eukaryotic genome.

**Table 1:** Comparison between our motifs and the three validated motifs reported in [17,6]

Our Motif	Validated Motif	Reference	Our PWM	Validated PWM	Similarity Score
GTGCAC	GTGCAC	PFF0200c[1]			0.997

GTGCAC shift 1b center extend 1b center	NGGTGCA	PfM18.1[2]			0.834

TATGCAT shift 2b right extend 1b right	TGCATGCA	PF14_0633[1]			0.704

### Motifs vs. gene function

We analyzed the distribution of the motif sets identified by our method within functionally relevant groups of genes. We used (1) clusters of genes inferred from GO annotations, (2) 15 previously published functional clusters derived from the analysis of mRNA profiles [21], and (3) a view of the previous 15 clusters reduced to four gene expression (GE) groups based on the morphological stage at which genes are expressed (see [21] and the Methods section). These GE groups were previously published [16] and correspond to clusters of genes grouped according to variations in FAIRE-seq within their promoters across seven time points. In the following we generically refer to any of these three sets above as a *functional gene set*. We estimated motif enrichment within each functional gene set using hypergeometric statistics.

**Figure 5:**
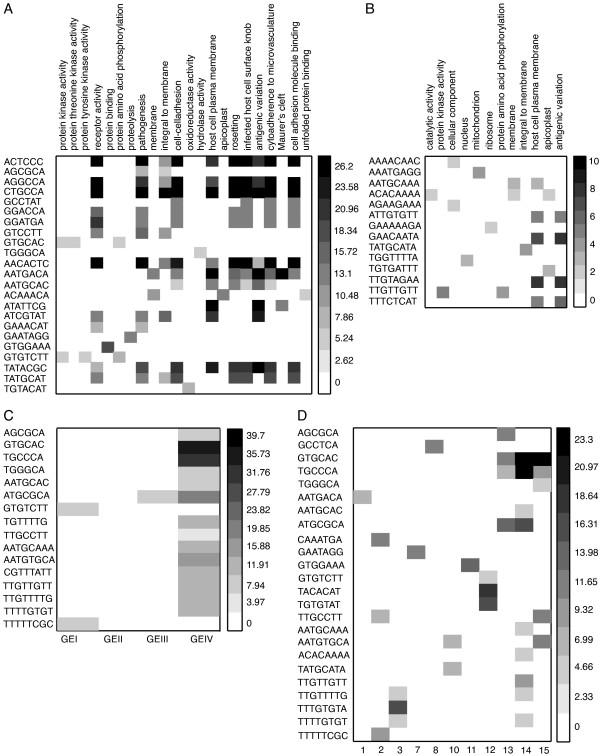
**Motif enrichment analysis**. A: GO-functional gene sets for motifs of length 6 and 7 bases. B: GO-functional gene sets for motifs of 8 bases in length. C: GE-groups. D: Gene expression-based clustering [21]. The darkness of each cell is proportional to the *z*-score of the corresponding motif in that functional gene set.

For each motif in our list we calculated the HES in a functional gene set by counting the number of occurrences of that motif within nucleosome-sized functional windows. Statistically significant motifs were selected using a randomized analysis. For each motif, 100 random gene clusters were generated and HES were computed for motifs within their functional windows. In order to assess statistical significance, a z-score was calculated using the average and standard deviation of the HES distribution for the random clusters of the same size of the functional gene set. We considered a motif in a functional gene set to be statistically significant if the *p*-value associated with the HES and the *p*-value associated with the z-score were both lower than 0.01 (HES > 2). Figure 5 show the z-scores of motif enrichment in GO-based clusters, the gene expression-inferred 15 functional clusters from [21], and the related GE functional gene sets proposed by Ponts *et al *[16]. Observe that motifs are either not found within a functional gene set or many of them are found. Increasing the length of the motif to 8 bp seems however to increase the specificity of the motif repartition by comparison with 6 and 7 bp-long motifs (Figure 5.A and 5.B). Here, we are facing the usual trade-off sensitivity *vs*. specificity, shorter *k-*mers allowing to discover more degenerated motifs but generating more false positives than longer ones with limited performances on motif variants. With regards to gene expression profiles-inferred functional gene sets, motifs are abundantly found at GEIV whereas none was observed for genes within GEII (Figure 5.C). Similar results are observed when all 15 functional clusters are considered (Figure 5.D). GEIV contains genes mostly involved in specialized stages of the parasite, *i.e.*, invasive, sexual, and mosquito stages. GEII consists of genes mostly involved in the asexual non-invasive forms of the parasite. The abundant presence of TFBS in GEIV gene promoters may reflect a highly regulated pattern of gene expression in specialized stages of the parasite. In contrast, the scarcity of motifs in the promoters of genes expressed during the asexual non-invasive stages of the parasite is consistent with the hypothesis of broad expression of genes without the accurate intervention of specific transcription factors to modulate transcription efficiency. These observations are in agreement with previous studies showing (1) significant discrepancies between mRNA levels and transcription rates [22], and (2) major regulatory mechanisms in the human malaria parasite are chromatin dynamics [16] and post-transcriptional regulations (such as mRNA decay) [23]. In summary, the enrichment analysis shows that the motifs identified by our chromatin structural change analysis via FAIRE-seq approach are likely to be biologically meaningful.

### Positional bias of the motifs relative to TSS and the predicted promoters

**Figure 6:**
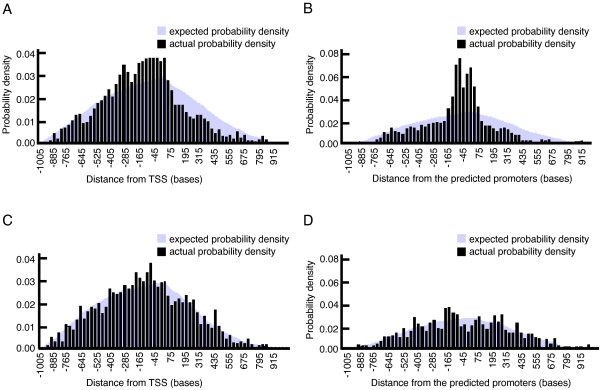
**Positional bias analysis**. A: our motifs relative to transcription start sites; B: our motifs relative to the predicted promoters; C: motifs proposed in [6] relative to transcription start sites; motifs proposed in [6] relative to the predicted promoters.

We conducted an analysis of positional bias relative to the transcription start site (TSS) and the predicted promoters reported in [24-26]. We first obtained the list of 2084 annotated TSS and 1027 predicted promoters, the latter given as a single genomic position. Then we calculated the number of occurrences of our motifs within a 2000 bp window centered at the TSS or the predicted promoter. Figures 6.A and 6.B show the distribution of positional occurrences of our motifs relative to TSS and predicted promoters, respectively. For comparison purposes, we computed the distribution of expected positional occurrences for our motifs if they were uniformly randomly distributed under an i.i.d. model (individual nucleotide frequencies were directly inferred from the *P. falciparum *genome composition). The observed probability density is shown in black and the expected in purple. As a preliminary observation, the presence of peaks in the observed probability density surrounding aligned TSS and predicted promoters suggest that some of our motifs have a strong positional bias relative to TSS and predicted promoters. The same analysis was carried out for the 50 candidate motifs proposed in [6] (Figures 6.C and 6.D) and the statistical significance of any positional bias was investigated as previously proposed [27].

Briefly, for each motif we computed the distribution of its positional occurrences within 5, 10, 15, 20, 25 and 30 bp-long sliding window (1) inside a region [-1000, -301] upstream of the TSS or the predicted promoter (*background*) and (2) inside a [-300, +300] region centered at the TSS or the predicted promoter. Using the positional distribution of the motif in the background, we computed its *p*-value to test the null hypothesis that the total number of occurrences of the motif inside a sliding window of the region centered at TSS (or predicted promoter) follows the distribution of the motif's occurrences inside a corresponding-size window in the background. We considered the number of occurrences of a motif inside a *k*-bases window (*k *= 5, 10, 15, 20, 25 and 30) to be statistically significant if its *p*-value was at most 0.01. Table 2 shows the percentage of our motifs that showed statistically significant positional bias relative to TSS. For comparison, we also provide the same analysis for the 50 motifs from [6] that showed statistically significant positional bias to TSS. Observe that the percentage of motifs with positional bias towards TSS are overall stronger for our motifs sets than for the set proposed by Young *et al*. [6]. This positional preference seems to be also stronger with regards to the predicted promoters. These results indicate that using FAIRE-seq profiles-based clustering for motif discovery could produce a more meaningful set of motifs than gene expression-based approaches.

**Table 2:** Percentage of motifs with positional bias relative to the TSS

Window width [bases]	Percentage of our motifs having *p*-value of 0.01 or lower	**Percentage of motifs in **[6]**having *p*-value of 0.01 or lower**
5	90.9%	88.0%
10	80.0%	76.0%
15	64.1%	72.0%
20	61.9%	52.0%
25	53.7%	52.0%
30	40.3%	44.0%

**Figure 7:**
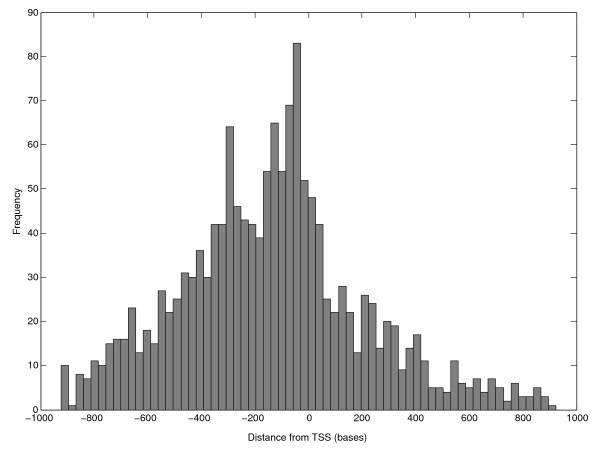
**Functional windows positional analysis**. The frequency histograms illustrates the distribution of positional preference of functional windows relative to transcription start sites.

Although the functional windows are chosen within 1000 bases upstream of the first codon independent of the TSS positions, we investigated whether the strong positional bias of our motifs relative to the TSS was influenced by the locations of the functional windows. Regions with the highest variance of FAIRE-seq coverage are expected to correlate with the position of the TSS, due to the coupling between the patterns of nucleosome occupancy and positioning preference of the transcription initiation complex. The positional distribution of the centers of the functional windows relative to TSS confirmed this hypothesis (Figure 7). There are two factors that contribute for the positional enrichment of motifs with respect to the TSS: the locations of the functional windows relative to TSS and the positions of the motif within the functional windows.

## Conclusion

The human malaria parasite has highly skewed nucleotide distribution (~80% of A+T) that together with the fact that over 40% of all genes have no known function makes *in silico *TFBS discovery challenging. Here we have demonstrated that chromatin structure data can be used in the context of an enumerative motif approach for successful *in silico *discovery of regulatory elements.

In our previous analysis of the dynamic chromatin structure in the human malaria parasite [16] we observed that FAIRE-seq coverage surrounding a validated TFBS varies drastically throughout the erythrocytic cycle. Another important observation was that most of the genes had well-defined FAIRE-seq peaks within their promoters, and while the intensity of the peaks changed across the time points, their locations relative to the start codons remained unchanged. Given these observations, we formulated the hypothesis that the most likely regions containing TFBS are the windows within the promoters with the highest variance in the FAIRE-seq coverage.

We tested this hypothesis by developing a new methodology for *in silico *motif discovery using FAIRE-seq coverage. Our approach resulted in the finding of 129 putative motifs, including many of the motifs proposed by previous studies [17,6,19,20]. Moreover, half of the motifs that we propose are over-represented within particular functional gene sets, especially genes involved in specialized stages of the parasite such as its sexual or invasive form. Our motifs also showed stronger positional bias relative to the promoter region compared to previously proposed set of motifs. These putative motifs together with their associative target clusters can serve as starting points in future research on characterization of unannotated proteins and regulatory mechanisms. In summary, our data confirm the importance of chromatin structural changes to regulate gene expression in the human malaria parasite. Precise knowledge of gene regulation pathways in the human malaria parasite will be essential for developing novel therapeutic strategies.

## Methods

### FAIRE-seq read processing and functional window selection

FAIRE-seq and alignment data were obtained from [16]. Using sequenced reads that align uniquely to the *P. falciparum *reference genome (downloaded from http://www.plasmoDB.org, version 5.5), FAIRE-seq read coverages were computed by extending the mapped reads up to 200 bases (*i.e.*, the average size of the sequencing libraries) as previously described [28]. The raw counts were then added at each position of the genome, and normalized per million of mapped reads and per percentage of area covered (see [16]). The functional window of a gene was identified in a 1000 bp-long region upstream of the start codon was identified as follows. The average FAIRE-seq coverage inside a sliding 146 bp-long (nucleosome size) window was computed for each region and for each time point. The window with the highest variance of average FAIRE-seq coverage across the seven time points was declared to be the *functional window *for that gene. The seven average values of FAIRE-seq coverage in the functional windows were used to generate the initial *k-*means clustering of the genes

### k-means clustering of coverage profiles of the functional windows

Clustering via k-means was performed using the FAIRE-seq coverage profiles in the functional windows of all protein-coding genes. Clustering was performed for *k *= 5, *k *= 10, *k *= 15, and *k *= 20. The k-means procedure for the initial clustering by FAIRE-seq coverage was carried out several times and always resulted in the same final assignment of genes to clusters. The underlying metric was Euclidean. Patterns for putative TFBS were searched for, using the clusters generated for each choice of *k*. The sets of motifs for each *k *were compared using Tanimoto distance and Pearson correlation coefficient. With a threshold on the Tanimoto distance of 0.5 and Pearson coefficient of 0.6, the sets of motifs according to various values of *k *were very similar. The choice *k *= 15 seemed to offer the best tradeoff between cluster size and the quality of cluster separation (data not shown). We therefore retained *k *= 15 in the rest of our analysis.

### Motifs scoring using a hypergeometric probabilistic model

All possible *k*-mers of length six to eight bases were searched within the functional windows of each gene. Frequencies of each *k-*mer were then modelled according to a hypergeometric distribution. For each cluster of genes, let *M *be the set of all *k-*mers inside the functional windows of the genes in the cluster. Let *n *denote the size of *M *and *y *denote the number of occurrences of a *k-*mer *m *in *M*. The frequency of each *k-*mer *m *of *M *was compared to its frequency observed in the set *S *of all *k-*mers found within 1000 bp upstream of genes (which also includes the defined functional windows). Let *N *denote the size of *S *and *r *denote the number of occurrences of *m *in *S*. The hypergeometric distribution was used to measure the probability that the frequency of a given *k-*mer of *M *would be observed if the input sequences would have been selected randomly within the entire promoter regions (1000 bp upstream of genes) rather than functional windows (see [29] for more details). For each *k-*mer *m *of *M*, a hypergeometric *p*-value was defined as:

$$P\left(N,r,n,y\right)=\overset{\text{min(}n\text{,}r\text{)}}{\underset{i=y}{\sum }}\frac{\left(\begin{array}{c} r\\ i \end{array}\right)\left(\begin{array}{c} N-r\\ n-i \end{array}\right)}{\begin{array}{c} N\\ n \end{array}}$$

The corresponding *hypergeometric enrichment score *(HES) is then defined as:

$$HES=-\log_{10}P\left(N,r,n,y\right)$$

The HES measures the statistical significance of a motif *m *in the positive set *M*. The smaller is the hypergeometric *p*-value, the smaller is the probability that the observed number of occurrences is due to random factors, and the higher is the HES.

### Motif identification and representation

Given a *k-*mer *t*, let *N*(*t*,1) denote the one-mismatch neighborhood of *t*, *i.e.*, a *k-*mer *s *belongs to *N*(*t*,1) if the number of mismatches between *s *and *t *is at most one. Given any *k-*mer *t *in the set *M*, the *k-*mers in *N*(*t*,1) were first sorted in decreasing order of their HES, and then a dynamic programming algorithm was used to select the set of *k*-mers in *N*(*t*,1) that maximized the HES. The HES for a set of *k*-mers is calculated using the formula in the previous section where *r *is the total number of occurrences of all *k*-mers in the set *S *and *y *is the total number of occurrences of all *k*-mers in the set *M *(*M *and *S *are also defined above). The dynamic programming algorithm works as follows. Let *L *be the ordered list of *k*-mers from *N*(*t*,1) by decreasing order of their HES. Each *k*-mer in *L *is considered one at a time in that order starting with *L*[1].We use *LN*[*i*] to denote a subset of top *i k*-mers in *L *whose overall HES is maximized over all possible such subsets that include the *i*^th ^*k*-mer, where 1 ≤ *i *≤ min{10, |*L*|}. Let *MH*[*i*] denote the HES calculated over *k*-mers in *LN*[i]. The recurrence relation for *MH*[*i*] is

$$MH\left[i\right]=\left\{\begin{array}{cc} HES\left(L\left[1\right]\right) & if\;i=1\\ \max\left\{HES\left(L\left[i\right]\right),S\left[i\right]\right\} & if\;i>1 \end{array}\right)$$

Where *S*[*i*]=max_1≤*j*<*i*_*HES*(over all k-mers in *LN*[*j*] and *L*[*i*]). Observe that *LN*[1] has only one *k*-mer, namely *L*[1]; hence, *MH*[*1*] is simply HES(*L*[1]), and our claim that HES over *k*-mers in *LN*[1] is maximized for top one *k*-mers is obviously true. To choose *LN*[*i*], we consider each *LN*[*j*] one at a time for all choices of 1≤*j*<i: we calculate HES over previously chosen *k*-mers in *LN*[*j*] together with *L*[*i*], and choose the *k*-mers in *LN*[*j*] ∪ *L*[*i*], whose HES is maximum over all choices for *j*. Then we compare the resulted maximum HES with HES of just one *k*-mer, namely *L*[*i*]: if HES(*L*[*i*]) is greater, then the final choice for *LN*[*i*] is *L*[*i*], otherwise *LN*[*i*] = *LN*[*j*]∪*L*[*i*]. Since *LN*[*j*] (for 1≤*j*<i) is a subset of top *j k*-mers that maximizes HES over all such subsets that include *j*^th ^*k*-mer, our choice of *LN*[*i*] increases the likelihood that it is a subset of top *i k*-mers that maximizes HES over all such subsets that include *i*^th ^*k*-mer. After the *MH *vector is calculated, the value of *i *corresponding to max*MH*[*i*] is determined and the set of *k*-mers in *LN*[*i*] becomes the mutant set of *t*. Observe that when we compute HES over *L*[*i*]∪*LN*[*j*], only *r *and *y *are affected in the formula for HES (both are increased). While this algorithm does not guarantee to always find the optimal subset of mutants with the lowest *p*-value, it works very well in practice and significantly decreases the computation time compared to the brute force approach.

Each *k*-mer *t *in *M *was represented by this HES-maximal subset that is called the *mutant set *of *t*. Since in practice the size of the mutant set is very small (less than ten), we only considered the top ten *k-*mers with the highest HES in *N*(*t*,1). To ensure that the selection of motifs followed a probabilistic model without replacement, we did not consider motifs whose occurrences of corresponding mutants overlapped each other.

For all identified motifs and their target gene clusters, we applied a final pipeline of filtration steps described next aimed at reducing the false positives.

### Motif identification and target genes clustering

Occurences of each exact *k-*mer were counted inside the 1000 bp promoter windows. The probability of occurrence of each *k*-mer was calculated under an independent and identically distributed (i.i.d.) model using individual nucleotide frequencies inferred from the genome composition. For each group of genes obtained by *k-*means clustering based on FAIRE-seq information, we selected potential *k-*mers for further analysis as follows. Given a *k-*mer *t *in the set *M *of all *k-*mers found within functional windows (see Motifs Scoring), first its one-mismatch neighborhood *N*(*t*, 1) was computed. A *k*-mer *t *was selected if (1) *t *occured in at least five distinct input sequences in a given cluster and (2) the expected number of distinct sequences in which any *k-*mer from *N*(*t*, 1) occured was smaller than the actual number of distinct sequences with *k-*mers from *N*(*t*, 1). Regarding the first condition, we chose to require five distinct input sequences because the smallest preliminary cluster was 33, and square root of 33 is approximately 5 [1]. The expected number of occurrences and the expected number of distinct sequences were computed on the positive set *M *of *k-*mers within the given sequences of a cluster of genes. Let *i *be a *k-*mer from *N*(*t*,1), then the expected number of occurrences of *i *in the positive set *M *is $\mu_{occ}^{i}=p_{i}n$, where *p_i _*is the probability of one occurrence of *i *in the genome, and *n *is the total number of *k-*mers in the positive set *M*. Then the expected number of distinct sequences containing *i *is

$$\mu_{seq}^{i}=1-e^{-\mu_{occ}^{i}}$$

and the expected number of distinct sequences for a motif *t *can be calculated as

$$\mu_{seq}=G\underset{i\in N\left(t,1\right)}{\sum }\mu_{seq}^{i},$$

where *G *is the number of sequences in the positive set *M*.

The first threshold on the minimum number of distinct sequences in which *k-*mers from *N*(*t*,1) must occur has the effect of filtering out motifs that have low probabilities of being over-represented in the positive set *M*. The second threshold filters out motifs that are abundant in the genome in general, and therefore an over-representation of these motifs in the positive set *M *is more likely random.

Each identified potential motifs was then analyzed in the context of their respective FAIRE-seq based preliminary cluster. For each *k-*mer *t*, we found the subset of mutants in *N*(*t*,1) that maximizes HES for *t *in the cluster. Then, we identified all genes that had exact occurrences of the mutants of *t *within the selected nucleosome-sized windows. These genes constituted a new *expanded *cluster for which the set of mutants for *t *was recomputed. This final set of mutants is a putative motif and the expanded cluster is its *target gene cluster*. Since the only three validated motifs for *P. falciparum *are of length 6 and 8 bases, we restricted our analysis to motif lengths 6 to 8 bases.

### Additional filtration steps

Since we calculated the HES based on the number of mutant occurrences rather than over the number of distinct genes where each mutant occurs, additional filtration steps are necessary to ensure that a high HES is not due to multiple occurrences within a few genes of the cluster. We required the number of distinct genes where the motif occured to be close to the number of occurrences of the motif in the target genes cluster. More specifically, we filtered out motifs whose ratio between the number of distinct genes where the motif occured and the number of occurrences of the motif in the cluster was smaller than 0.8. Since the HES of a motif was calculated over sequences of about 150 bp in length, we wanted the number of exact occurrences of mutants inside a single sequence to be close to one. This threshold was used to distinguish between motifs and tandem repeats. Finally, we only considered motifs with HES in their target clusters greater than or equal to the average HES in the distribution of HES for all motifs of length 6 to 8 bases.

### Phylogenetic conservation analysis

We used phylogenetic conservation information to filter out non-conserved motifs that are likely to be false positives. Similarly to [6], we calculated HES for the putative motifs in four orthologous species, namely *P. berghei*, *P.chabaudi*, *P. vivax *and *P. yoelii*. For each putative motif, we computed the HES for genes orthologous to the genes in the motif's target cluster within 1000 bp upstream of the start codon. Motifs with a HES of at least 2 (*i.e.*, *p*-value of 0.01) in at least one of the four orthologous species were kept for further analysis. To avoid artificially high orthologous HES, we required the ratio between the number of distinct genes where the mutants occurred and the number of exact occurrences of motifs in the set of orthologous genes corresponding to the target cluster to be at least 0.5 (this threshold is lower than in the previous step due to the increased length of input sequences). This step ensured that HES was supported with enough orthologous genes and that all occurrences did not fall into the promoters of a few genes.

To confirm that the statistical significance of our motifs in their corresponding orthologous clusters are not due to a random chance, we conducted an additional randomized analysis. Particularly, we investigated the distribution of HES calculated for our motifs in randomly chosen orthologous clusters. For each motif we calculated its HES in 100 randomly chosen orthologous clusters of the same size that was used in our original analysis. Then we calculated the average and the standard deviation of HES over the random clusters and used these values to find a new threshold on the orthologous HES corresponding to a significance level of 0.01. A motif was kept for further analysis if it had orthologous HES of at least two and this HES was higher than the threshold calculated over the random orthologous clusters. This analysis ensures that orthologous HES is statistically significant for each motif and the given cluster size.

Finally, we adjusted the orthologous *p*-values of each final motif to correct for multiple testing. Our permutation test gives us the distribution of the orthologous HES when the enrichment of a motif in an orthologous cluster is solely due to a random chance. For each enriched motif, we computed its HES in 1000 randomly-selected orthologous clusters in the four orthologous species, where the size of a random orthologous cluster was the same as the size of the original orthologous target gene cluster. Since the statistical significance test requires that the orthologous HES must exceed the threshold in at least one out of four species, we computed the HES in all four species at each iteration, but used in the analysis only the maximum score. In order to be able to compare HES across different motifs (which might have different cluster sizes), we normalized all HES (both for real and random clusters) using their mean *μ_HES _*and the standard deviation *σ_HES_*, calculated over 1000 random clusters. The normalized HES is (HES -*μ_HES_*)/*σ_HES_*. The resulting distribution of 1000N normalized HES (where N is 1587, 2471, and 2745 for 6-, 7-, and 8-mers respectively), was used to correct for multiple testing, as follows. The normalized HES for both real and random clusters were aggregated, then sorted in decreasing order. For each final motif we calculated the *p*-value (corrected for multiple testing) as the proportion of the top values in this joint distribution that are greater than or equal to the real orthologous normalized HES of the motif [30]. Out of the final 129 motifs reported previously, a total of 113 have adjusted *p*-values corresponding to a false discovery rate of less than 5%. Additional File 1 contains the *p*-values for all 129 motifs before and after correction for multiple testing.

### Motifs clustering

In order to account for sequence degeneration of motifs we added a motif clustering step to filter out duplicates and highly similar motifs. We selected unique motifs with the highest HES and clustered the remaining putative motifs by similarity using the *Tanimoto *distance [3,6] and Pearson correlation coefficients of their position weight matrices (PWMs) [18].

The Tanimoto distance measures pairwise similarity of the motifs. Motifs with pairwise distances smaller than 0.5 were grouped, and the one with the highest HES was selected to represent the group. In other words, if two motifs shared at least half of their positions then they belonged to the same group. Since our method does not rely on co-regulated clusters, we assumed that for a given motif not all genes in the target genes cluster were co-regulated. That is why we allowed a lower threshold for the Tanimoto distance by comparison with the threshold of 0.8 used [3,6]. Given the positional occurrences for two motifs represented in sets *A *and *B *respectively, we defined two occurrences (one from *A *and one from *B*) to be *overlapping *if they shared at least one base. The Tanimoto distance was calculated as follows

$$1-\frac{\left|A\cap B\right|}{\left|A\cup B\right|}$$

where the intersection of *A *and *B *includes all overlapping positional occurrences.

Then, motifs were clustered according to the Pearson coefficients of their PWMs. This step filtered out motifs that had very similar content, *e.g.*, motifs that might be shifted versions of each other. We define two motifs *shifted version *if they shared at least half of a motif's length. We considered two motifs *similar *if the Pearson coefficient between their corresponding PWMs or of the PWMs built on their shifted versions was greater than or equal to 0.75 [7,18]. To build PWMs for two motifs that are shifted versions of each other, we first built a PWM for the motif with the highest HES and then we constructed a PWM for the other motif that we shifted to align with the highest-HES-motif.

### Gene orthology and gene functional sets

Orthologous gene maps between *P. falciparum *and *P. berghei*, *P. falciparum *and *P. chabaudi*, *P. falciparum *and *P. vivax*, and *P. falciparum *and *P. yoelii *were obtained from the OrthoMCL database http://www.orthomcl.org/]. A total of 1915 orthologous genes of *P. berghei*, 1247 of *P. chabaudi*, 4126 of *P. vivax*, and 2685 of *P. yoelii *were used in this study.

In order to study the motifs enrichment in functional gene sets, we used gene ontology (GO) functions from *P. falciparum *v6.0 together with ontology-based pattern identification (OPI) clusters from [http://carrier.gnf.org/publications/OPI/] to retrieve a total of 13,859 GO/gene pairs, with 1288 distinct GO names. As an alternative for GO annotation, we used 15 functional clusters of genes that were previously obtained based on mRNA profiles [21]. These 15 clusters were used as is or regrouped into four gene expression (GE) groups as follows. Gene expression group I (GEI) contains genes expressed in sporozoites and gametocytes (clusters 1-3), GEII contains genes corresponding to ring, schizont and trophozoite stages (clusters 4-7), GEIII contains genes expressed at the throphozoite stage (clusters 8-13) and group GEIV contains genes expressed at sporozoite, gametocyte and schizont stages and involved in red blood cell invasion (clusters 14-15).

## Authors' contributions

KLR and NP designed the study, provided the FAIRE-seq data. EYH designed the algorithm, implemented the software tool, carried out the experiments, and wrote the initial draft of this manuscript. KLR, NP and SL supervised the project and edited the manuscript. All authors read and approved the final manuscript.

## Supplementary Material

Additional file 1 **File Additional_Table1.xls is an Excel spreadsheet. The list of 129 motifs identified by our method**. **Column A: **ID of the FAIRE cluster for this motif **Column B: **k-mer that was used to find 1-mismatch (2-mismatch) neighborhood **Column C: **obtained by aligning the selected set of mutants for a motif **Column D: **another representation of the consensus of aligned mutants **Column E: **total number of mutants for a motif **Column F: **number mismatches allowed for a motif **Column G: **number of distinct genes, with occurrences of mutants within the functional windows (colors) **Column H: **total number of occurrences of mutants within functional windows of the target gene cluster **Column I: **total number of occurrences of mutants within 1000 bases promoters **Column J: **size of the target gene cluster (Note: color <= cluster size, because target gene cluster is selected for 1-mism neighborhood, but color is identified for the selected set of mutants that maximize HES) **Column K: **Hypergeometric Enrichment score (HES) = -log(p-value) **Column L: **HES in orthologous pbe **Column M: **HES in orthologous pch **Column N: **HES in orthologous pyo **Column O: **HES in orthologous pvivax**Column P: **Maximum value of pbe-hes, pch-hes, pyo-hes, pvivax-hes **Column Q: **Minimum p-values out of pbe, pch, pyo, pvivax (corresponding to max-ortholog HES), before correction for multiple testing **Column R**: Adjusted p-value corrected for multiple testing. Click here for file
